# Navigating Challenges in Cervicofacial Actinomycosis: A Case of Diagnostic Persistence and Multidisciplinary Collaboration

**DOI:** 10.7759/cureus.68533

**Published:** 2024-09-03

**Authors:** Sandra Sony, Sarath C Sistla, Mrudula Rao

**Affiliations:** 1 General Practice, ASHWINI (Association for Health Welfare in the Nilgiris) - Gudalur Adivasi Hospital, Gudalur, IND; 2 General Surgery, Vivekananda Memorial Hospital, Sargur, IND; 3 Family Medicine, ASHWINI (Association for Health Welfare in the Nilgiris) - Gudalur Adivasi Hospital, Gudalur, IND

**Keywords:** osteomyelitis, multidisciplinary approach, splendore-hoeppli phenomenon, cervicofacial, actinomycosis

## Abstract

Actinomycosis is a rare, chronic bacterial infection caused by *Actinomyces* species, characterized by granulomatous inflammation, abscesses, and sinus tracts. It primarily affects the cervicofacial region and often mimics other conditions such as malignancies or tuberculosis, complicating early diagnosis and treatment. This case report details an 18-year-old male with no known comorbidities, who presented with a two-week history of facial swelling, trismus, and discharging fistulas following an insect bite. The delay in seeking medical attention was due to initial symptom subsidence and challenges in accessing care. Diagnostic difficulties arose from inconclusive pus cultures and the lack of specialist resources at our facility. An incisional biopsy and subsequent histopathological examination revealed basophilic Gram-positive, non-acid fast filamentous bacteria and the Splendore-Hoeppli phenomenon, ultimately confirming actinomycosis. This case highlights the necessity of including actinomycosis in the differential diagnosis of facial infections and demonstrates the value of a multidisciplinary approach in managing complex cases with diagnostic and therapeutic challenges.

## Introduction

Actinomycosis is a relatively rare, chronic bacterial infection caused by anaerobic Gram-positive bacteria of the genus *Actinomyces*, typically found in the oral cavity and other mucosal surfaces [1,2]. Among its various clinical forms, cervicofacial actinomycosis is the most common, often originating from odontogenic infections or local trauma [3]. This condition presents significant diagnostic and management challenges because it can mimic other diseases such as malignancies or tuberculosis [1].

Cervicofacial actinomycosis usually manifests as progressive inflammatory swelling that may develop into purulent, draining fistulas [4]. Key precipitating factors include poor dental hygiene, recent oral surgery, and local tissue inflammation [1,5]. The condition is notably characterized by the presence of sulfur granules, which are crucial for distinguishing it from other infections [1,6,7].

Though less common, osteomyelitis of the jaw can occur secondary to actinomycotic infections, leading to severe symptoms such as trismus and swelling, which further complicate diagnosis and treatment [2,8]. This case underscores the importance of maintaining a high index of clinical suspicion, utilizing appropriate diagnostic methods, and adopting a combined approach of antibiotic therapy and surgical intervention to effectively manage this rare infection.

## Case presentation

An 18-year-old tribal boy with no known comorbidities presented on June 23, 2024, with a two-week history of swelling on the right side of the face and two small wounds with blood-tinged pus discharge, associated with difficulty opening his mouth. This was preceded by an insect bite on the face about seven days before the onset of a painful swelling in the affected area. He gave no history of atopy in the past. The pain eventually subsided over a week without any specific treatment, leading to a delay in hospital presentation. However, the associated trismus, which interfered with his ability to eat, prompted him to seek medical attention.

**Figure 1:**
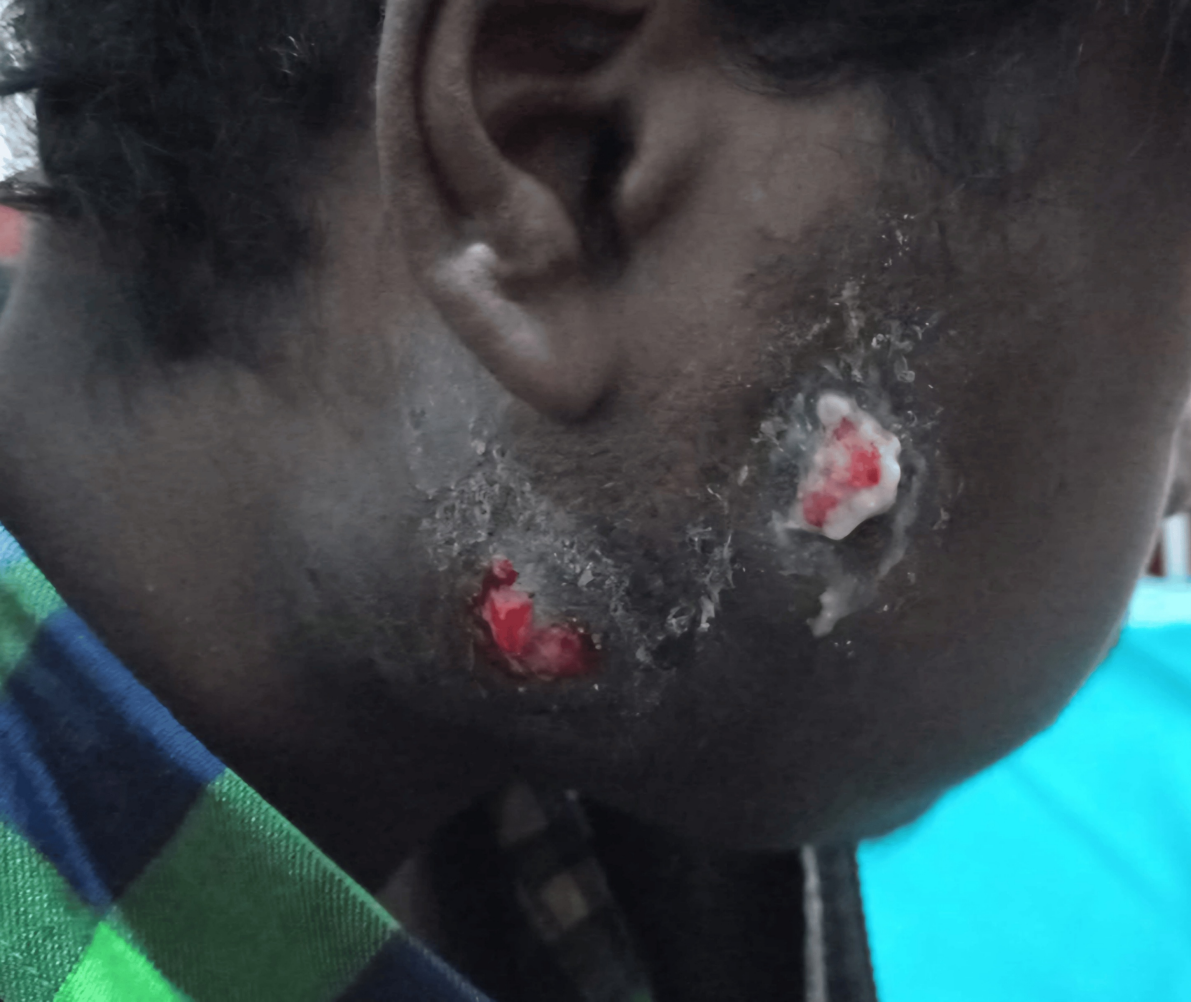
Image showing swelling of the entire right cheek with two discharging sinuses with blood-tinged purulent discharge.

On clinical examination (Figure 1), there was a firm-to-hard diffuse swelling involving the entire right side of the face, approximately 10 x 12 cm, with two 2-cm fistulae on the skin surface discharging minimal pus. The patient had severe trismus, with a mouth opening of about 1 cm. He was afebrile and his vitals were stable.

**Figure 2:**
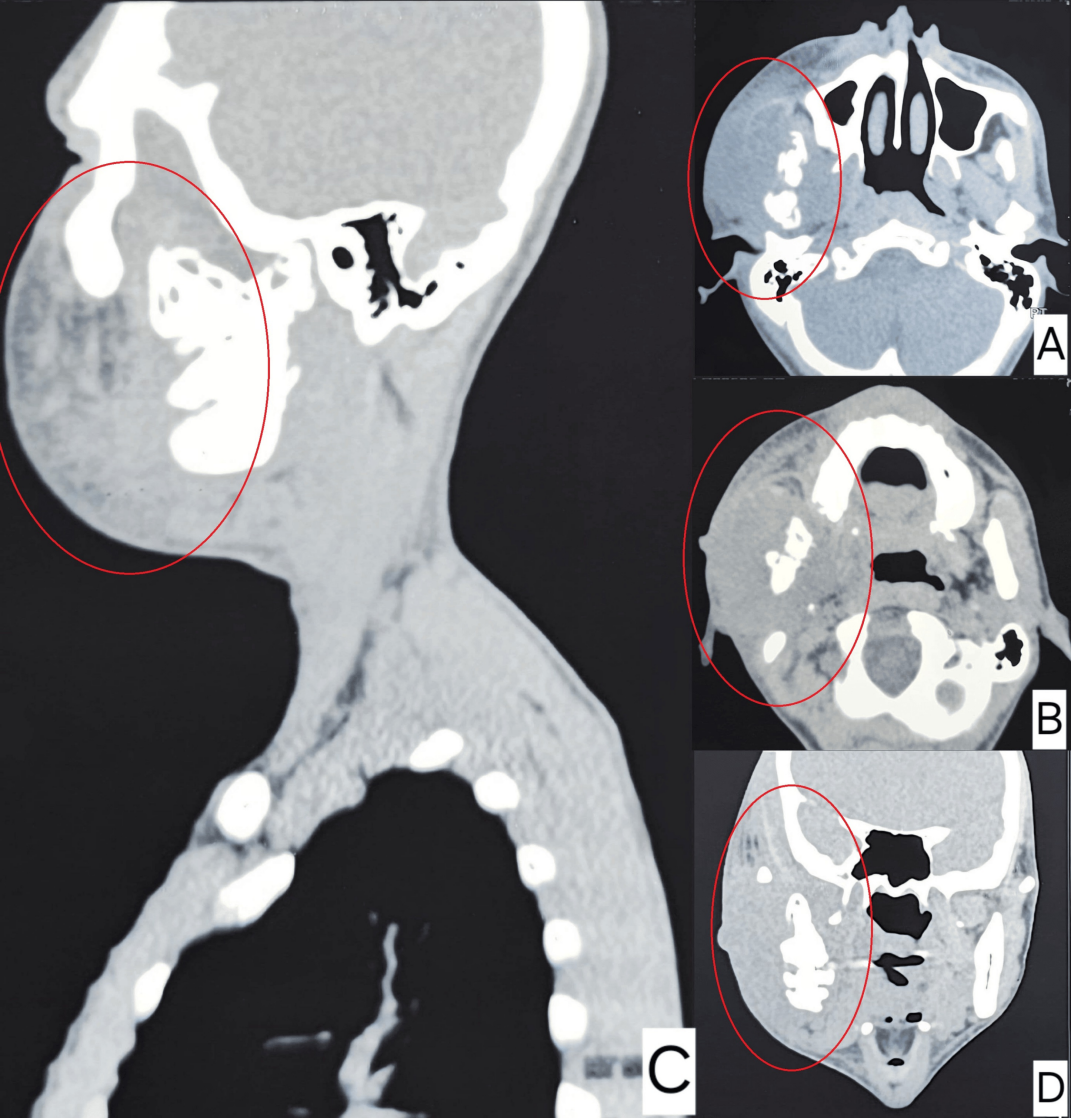
Computed tomography scan image showing heterogeneous soft tissue thickening with bone destruction involving the right ramus of the mandible and the right temporomandibular joint. Irregularly marginated heterogeneous soft tissue dense lesion noted around the ramus of the right hemimandible causing lysis with increased bone marrow attenuation involving the same. Few ill-defined hypodense areas were noted within, likely representing necrosis/evolving abscess. No calcification was noted (B, C, and D). Laterally, the soft tissue-dense lesion involves the master muscle reaching the skin surface. No definite focal defect in the skin surface suggesting fistula formation (B and D). Medially, involving both medial and lateral pterygoid muscles (B and C). Superiorly, extending into the infratemporal fossa with involvement of the infrazygomatic part of the right temporalis muscle and effacement of the retro maxillary fat pad (B). No significant cervical lymphadenopathy.

A swab from the lesions was sent for Gram stain examination and bacterial culture with sensitivity testing. Basic blood investigations, including complete blood count (CBC), fasting blood sugar, random blood sugar, and urinalysis, were also performed. Initially, the patient was started on oral antibiotic therapy with amoxicillin + clavulanic acid 625 mg thrice daily, based on a provisional diagnosis of a bacterial infection. A non-contrast computed tomography (CT) of the head and neck (Figure 2) revealed an irregularly marginated heterogeneous soft tissue dense lesion around the ramus of the right hemimandible causing lysis with increased bone marrow attenuation involving the same. Few ill-defined hypodense areas were noted within, likely representing necrosis/evolving abscess. However, the CT did not provide a definitive diagnosis. The pus culture report showed no growth, which led to a diagnostic dilemma. Despite five days of oral antibiotic therapy, there was no significant reduction in the swelling or improvement in the trismus.

Due to the non-availability of specialist care at our hospital and the lack of resolution of the disease with conventional antibiotic therapy, an orthopedic surgeon's opinion was sought telephonically, who suggested seeking an expert opinion from a maxillofacial surgeon for better clarity on further management and clearer diagnosis. After unsuccessful attempts to persuade the family to seek a referral to a higher center, a maxillofacial surgeon from the nearest hospital was requested to visit our facility for consultation and expert opinion regarding management.

Following the maxillofacial surgeon's advice, the management protocol was modified. The antibiotic therapy was changed to an intravenous route, with ceftriaxone 2000 mg once a day and metronidazole 400 mg every six hours. The ulcer sites were widened, and an attempt at drainage and curettage was made, revealing minimal pus discharge with extensive surrounding hard, indurated tissue. An incisional biopsy was performed and sent for histopathological examination.

**Figure 3:**
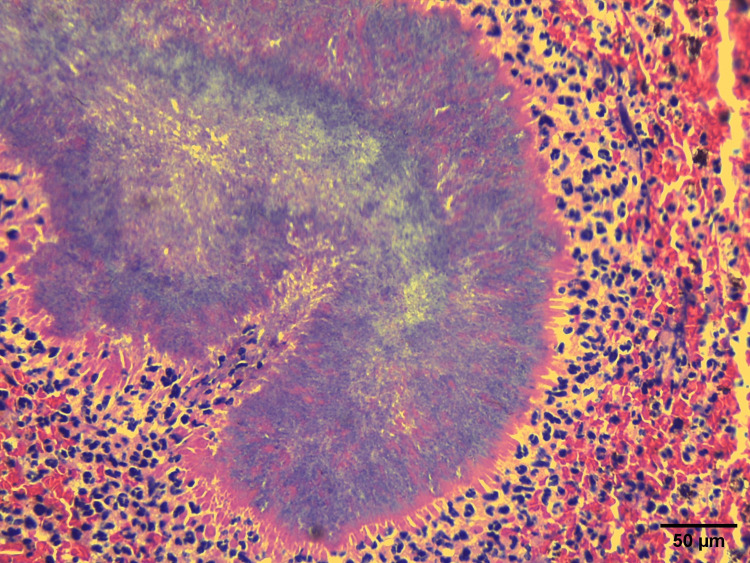
Incisional biopsy of the lesion. The section, stained with Grocott's methenamine silver (GMS) stain and viewed at 40x magnification, shows skin with fragments of granulation tissue exhibiting a dense neutrophilic infiltrate, histiocytes, and proliferating blood vessels. Focal areas display clumps of basophilic filamentous bacteria arranged in a vague rosette-like configuration. Eosinophilic clubs found at the periphery are suggestive of the Splendore-Hoeppli phenomenon. No evidence of granulomas or atypical cells is seen.

Microscopic examination (Figure 3) revealed skin fragments with granulation tissue, a dense neutrophilic infiltrate with histiocytes, proliferating blood vessels, and focal areas showing clumps of basophilic filamentous bacteria arranged in a vague rosette-like configuration. Eosinophilic clubs were found at the periphery, suggestive of the Splendore-Hoeppli phenomenon. Special stains revealed Gram-positive, filamentous bacilli that showed positivity on both Grocott methenamine silver (GMS) and periodic acid-Schiff (PAS) stains, and a negative Ziehl-Neelsen (ZN) stain, supporting the diagnosis of cervicofacial actinomycosis while ruling out nocardiosis and other fungal infections.

Daily dressings and continued IV antibiotics were administered for a total of 14 days. The patient was discharged on July 11, 2024, on oral amoxicillin + clavulanic acid 625 mg thrice daily. The patient was instructed to perform ice-cream stick exercises to improve mouth opening.

**Figure 4:**
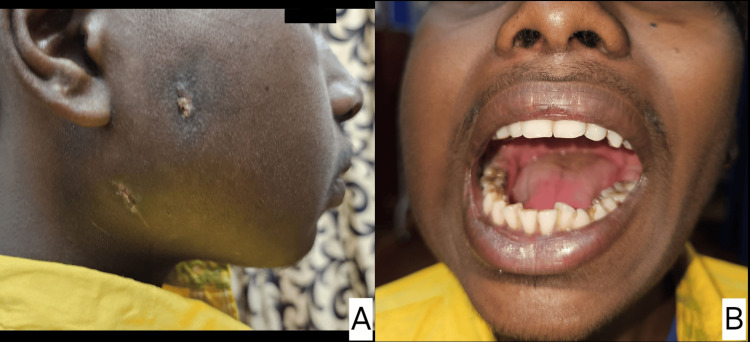
Image taken at the five-day follow-up. Figure A demonstrates a significant reduction in swelling and a noticeable closing up of the sinus tracts. Figure B illustrates an improvement in mouth opening, achieving a functional grade.

**Figure 5:**
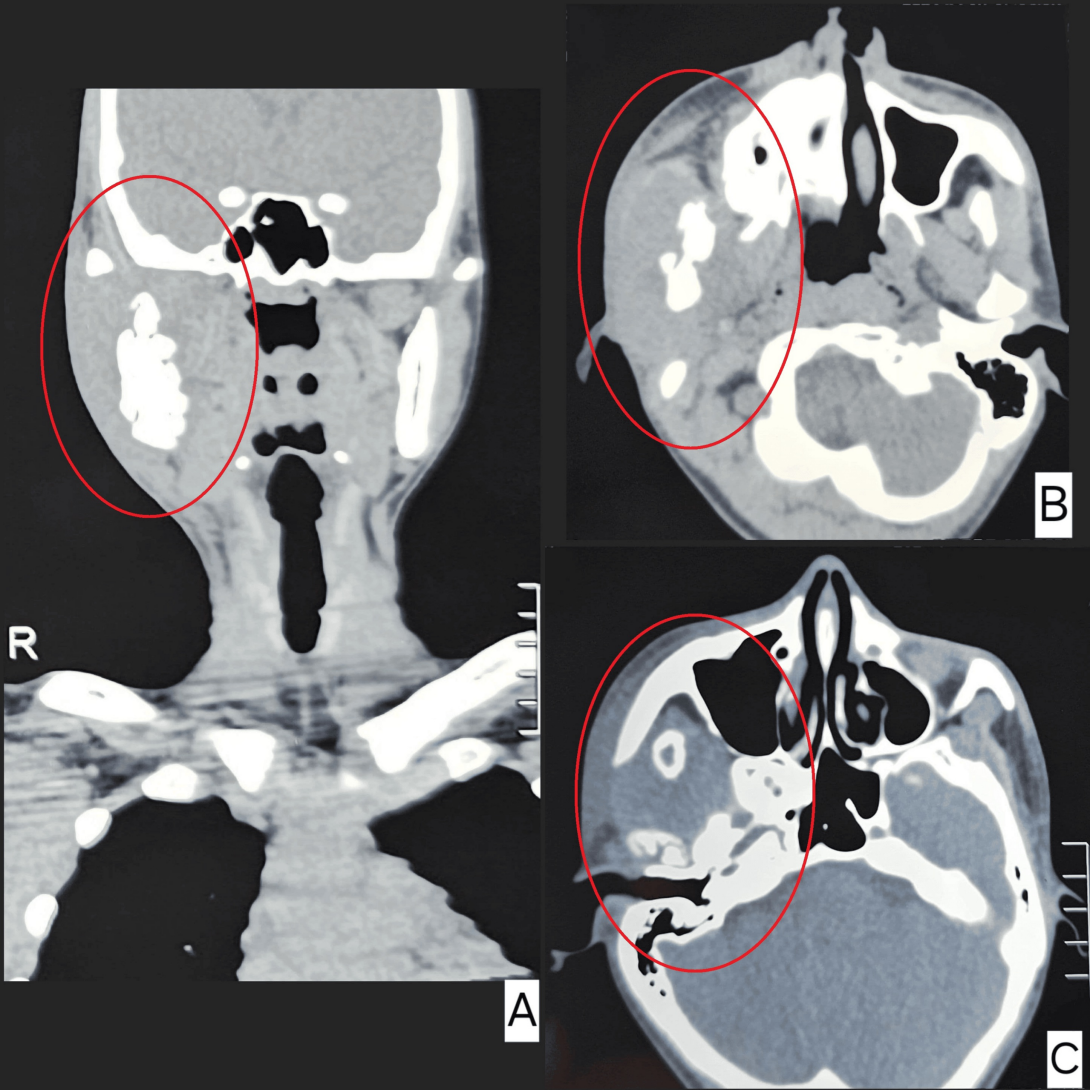
Computed tomography scan taken at the five-day follow-up. Interval decrease in irregularly marginated soft tissue dense lesion with persistent signs of adjacent bone inflammatory changes (A and B). No evidence of any ill-defined hypodense regions within the soft tissue dense lesion to suggest active abscess formation or ongoing necrotic changes (B). Significant resolution of adjacent soft tissue swelling, ipsilateral pre-maxillary swelling, and effacement of the retro maxillary fat pad (B and C). Interval decrease in involvement of infratemporal fossa and involvement of infrazygomatic part of right temporalis muscle - superiorly (C). Persistent signs of involvement in the masseter muscle and skin-laterally, medial and lateral pterygoid - medially, effacement of the ipsilateral para pharyngeal fat pad and likely causing abutment of adjacent torus tubarius with effacement of the ipsilateral eustachian tube (B and A). The above features are suggestive of a significant treatment response with an interval decrease in the soft tissue density lesion.

Significant clinical improvement (Figure 4) was noted at the five-day follow-up on July 16, 2024, with a marked reduction in swelling and improvement in mouth opening to a functional grade of three fingers. The patient reported greater comfort and improved ability to eat. The sinus tracts had closed, and a follow-up non-contrast CT (Figure 5) showed a mild reduction in the collection around the right ramus of the mandible and a significant periosteal reaction. The patient was continued on oral antibiotics for complete resolution of the condition and scheduled for monthly follow-ups to monitor disease resolution.

## Discussion

Diagnostic challenges in cervicofacial actinomycosis

Cervicofacial actinomycosis presents significant diagnostic challenges, primarily due to its rarity and its potential to mimic other conditions such as malignancies or tuberculosis [9]. In this case, the patient’s initial presentation, a two-week history of facial swelling and trismus following an insect bite, highlighted the complexities involved in distinguishing actinomycosis from more common facial infections or conditions. The initial treatment plan to modify the treatment regimen based on pus culture results, which unfortunately yielded no growth, further complicated the diagnosis. This aligns with findings from previous studies, which emphasize that traditional culture methods often fail to identify *Actinomyces* species due to their anaerobic nature and slow growth rate [6,10,11].

Histopathological examination became pivotal in this case, ultimately revealing the presence of basophilic filamentous bacteria and the characteristic Splendore-Hoeppli phenomenon [12,13]. This confirmation underscores the importance of considering actinomycosis in the differential diagnosis of chronic facial infections, especially when culture results are inconclusive. Previous literature on this type of clinical presentation supports this approach, indicating that histological identification remains a crucial component of diagnosis in challenging cases.

Treatment strategies and challenges

The initial oral antibiotics were ineffective, necessitating a switch to intravenous antibiotics, which is consistent with recommended practices for managing actinomycosis, particularly in cases with severe symptoms or complications [14]. The subsequent surgical intervention, including drainage and curettage, was essential for addressing the indurated tissue and residual infection.

The patient's clinical improvement following the modification of the treatment regimen reflects the necessity of adapting therapeutic strategies based on ongoing assessment and response. This aligns with the established treatment protocols for actinomycosis, which advocate for prolonged antibiotic therapy and surgical debridement in more severe or refractory cases [15].

Multidisciplinary approach

This case underscores the value of a multidisciplinary approach in managing complex infections. The involvement of an orthopedic surgeon and a maxillofacial surgeon was crucial in navigating both the diagnostic and therapeutic challenges. Collaboration between specialties facilitated a comprehensive treatment plan and addressed the complications effectively, highlighting a model for managing similar cases in resource-limited settings.

Resource limitations and implications

The case illustrates the difficulties encountered in a resource-constrained environment, where access to specialized diagnostic tools and expert consultations may be limited. The delay in definitive diagnosis and the reliance on histopathological examination due to the lack of specialist care emphasize the need for adaptability and resourcefulness in such settings. These challenges are reflective of broader issues identified in the management of rare infections in underserved areas.

## Conclusions

Cervicofacial actinomycosis should be considered in the differential diagnosis of chronic facial infections, particularly when initial diagnostic tests are inconclusive. The integration of histopathological examination and a multidisciplinary treatment approach, including prolonged antibiotic therapy and surgical intervention, is crucial for successful management. This case not only highlights the diagnostic and therapeutic challenges associated with actinomycosis but also underscores the importance of adapting treatment strategies to the clinical context and available resources.
